# Acculturation and Healthcare Utilization Among Older Korean Immigrants: A Dyadic Study of Married Couples

**DOI:** 10.1093/geroni/igaa057.1945

**Published:** 2020-12-16

**Authors:** Kyungmin Kim, Yuri Jang, Nan Sook Park, David Chiriboga

**Affiliations:** 1 University of Massachusetts Boston, Boston, Massachusetts, United States; 2 University of Southern California, Los Angeles, California, United States; 3 University of South Florida, Tampa, Florida, United States

## Abstract

Research has focused on the socioeconomic/cultural characteristics of individuals to address health disparities among immigrant populations. Dyadic studies of acculturation and healthcare utilization among older immigrants are rare. Using data from 263 older Korean immigrant couples in the U.S. (Mean_age = 74.75 for husbands; 71.03 for wives), this study examined how each spousal acculturation levels (e.g., English proficiency, familiarity with American culture) are associated with healthcare utilization (e.g., usual source of care, medical checkup) and difficulty in using health services, controlling for sociodemographic characteristics. Overall, husbands showed higher levels of acculturation than their wives, but there was also substantial similarity between spouses (ICC = .58). For healthcare utilization, one’s own acculturation (actor effect) was significant only for wives, but spouse’s acculturation (partner effect) was significant only for husbands. For difficulty in health service use, one’s own acculturation was significant for both spouses, but spouse’s acculturation was significant only for husbands.

